# Targeting NF-κB Signaling by Calebin A, a Compound of Turmeric, in Multicellular Tumor Microenvironment: Potential Role of Apoptosis Induction in CRC Cells

**DOI:** 10.3390/biomedicines8080236

**Published:** 2020-07-22

**Authors:** Constanze Buhrmann, Parviz Shayan, Kishore Banik, Ajaikumar B. Kunnumakkara, Peter Kubatka, Lenka Koklesova, Mehdi Shakibaei

**Affiliations:** 1 Musculoskeletal Research Group and Tumor Biology, Chair of Vegetative Anatomy, Institute of Anatomy, Faculty of Medicine, Ludwig-Maximilian-University Munich, Pettenkoferstrasse 11, D-80336 Munich, Germany; constanze.buhrmann@med.uni-muenchen.de; 2 Department of Parasitology, Faculty of Veterinary Medicine, University of Tehran, Tehran 141556453, Iran; pshayan@ut.ac.ir; 3 Cancer Biology Laboratory & DBT-AIST International Center for Translational and Environmental Research (DAICENTER), Department of Biosciences & Bioengineering, Indian Institute of Technology Guwahati, Assam 781039, India; kishore.banik@iitg.ac.in (K.B.); kunnumakkara@iitg.ac.in (A.B.K.); 4 Department of Medical Biology, Jessenius Faculty of Medicine, Comenius University in Bratislava, 03601 Martin, Slovakia; peter.kubatka@uniba.sk; 5 Department of Obstetrics and Gynecology, Jessenius Faculty of Medicine, Comenius University in Bratislava, 036 01 Martin, Slovakia; koklesova.lenka@gmail.com

**Keywords:** Calebin A, colorectal cancer, tumor microenvironment, NF-κB signaling, apoptosis

## Abstract

Increasing lines of evidence suggest that chronic inflammation mediates most chronic diseases, including cancer. The transcription factor, NF-κB, has been shown to be a major regulator of inflammation and metastasis in tumor cells. Therefore, compounds or any natural agents that can inhibit NF-κB activation have the potential to prevent and treat cancer. However, the mechanism by which Calebin A, a component of turmeric, regulates inflammation and disrupts the interaction between HCT116 colorectal cancer (CRC) cells and multicellular tumor microenvironment (TME) is still poorly understood. The 3D-alginate HCT116 cell cultures in TME were treated with Calebin A, BMS-345541, and dithiothreitol (DTT) and examined for invasiveness, proliferation, and apoptosis. The mechanism of TME-induced malignancy of cancer cells was confirmed by phase contrast, Western blotting, immunofluorescence, and DNA-binding assay. We found through DNA binding assay, that Calebin A inhibited TME-induced NF-κB activation in a dose-dependent manner. As a result of this inhibition, NF-κB phosphorylation and NF-κB nuclear translocation were down-modulated. Calebin A, or IκB-kinase (IKK) inhibitor (BMS-345541) significantly inhibited the direct interaction of nuclear p65 to DNA, and interestingly this interaction was reversed by DTT. Calebin A also suppressed the expression of NF-κB-promoted anti-apoptotic (Bcl-2, Bcl-xL, survivin), proliferation (Cyclin D1), invasion (MMP-9), metastasis (CXCR4), and down-regulated apoptosis (Caspase-3) gene biomarkers, leading to apoptosis in HCT116 cells. These results suggest that Calebin A can suppress multicellular TME-promoted CRC cell invasion and malignancy by inhibiting the NF-κB-promoting inflammatory pathway associated with carcinogenesis, underlining the potential of Calebin A for CRC treatment.

## 1. Introduction

Colorectal cancer (CRC) is the third most common cancer occurring globally and is among the five leading causes of deaths due to cancer in the world [1]. It is now recognized that CRC is a multifactorial process involving risk factors such as environment, lifestyle, and genetic predisposition [2,3] and that CRC is a multi-stage disease that is promoted by chronic, low-grade inflammation of the bowel [4].

Recent research has shown that inflammation in the tumor cells of CRC and its immediate surroundings, known as the TME, plays a crucial role in the malignant progression of CRC [5,6]. Here, under the influence of cytokines, growth factors, and chemotactic stimuli, the tumor cells recruit stromal fibroblasts and transform them into malignant ones and create an environment that is capable of supporting the progression of tumor malignancy [5,7]. As a matter of fact, the transformed stromal fibroblasts secrete tumor-stimulating factors and play a vital role in transforming the extracellular matrix to facilitate tumor invasion and metastasis [8]. In addition to the transformed stromal fibroblasts, immune cells, such as B- and T-lymphocytes, are also essential to promote tumor malignancy and evade the immunological response by producing reactive oxygen species and inflammatory cytokines, thereby stimulating inflammatory cascades in the cancer cells [9,10,11].

The transcription factor nuclear factor-kappaB (NF-κB) is a major regulator of inflammation and has been found to be frequently activated as a stress responder and stimulates low-grade chronic inflammation [12,13]. In its inactive form, NF-κB is found in the cytoplasm of various cells as homo- and/or heterotrimers with p50, p52, c-Rel, RelA (p65) and RelB subunits, and the activation of the pathway is divided into canonical and non-canonical signaling pathways [14,15]. The activation of canonical NF-κB signaling is strictly regulated by cytoplasmic inhibitory proteins of the IκB family [16,17], which in turn are regulated by IκB kinase (IKK) complexes (IKKα, IKKβ, IKKγ) [15,18].

NF-κB is crucial for biological processes that regulate cell growth, survival and tissue development [14]. It has been shown that the constitutive activation of NF-κB in cancer stimulates cancer cell progression [19,20,21]. Hereby, NF-κB is closely involved in the suppression of cellular apoptosis and mediates its actions through the up-regulation of anti-apoptotic genes such as B cell lymphoma extra-large (Bcl-xL), cellular apoptosis inhibitors (cIAPs), survivin, B-cell lymphoma (Bcl-2), X-linked inhibitor of apoptosis protein (XIAP), and caspase-8/FAS-associated death domain-like IL-1β converting enzyme inhibiting protein [15,22,23].

NF-κB activation has been shown to stimulate the inflammatory response of TME and promote cancer by supporting immune modulations, tumor cell survival, and paracrine signaling of pro-inflammatory cytokines in the TME [11,24]. Furthermore, the activation of autocrine and paracrine signals from members of the tumor necrosis factor (TNF) family has a major impact on invasion and metastasis of cancer cells [25,26,27,28]. Studies have shown that lymphocytes that secrete TNF-β into the TME stimulates cancer progression by activation and interaction via the TNF-receptor/NF-κB signaling pathway [29]. Therefore, targeting the NF-κB signaling pathway has also revealed to be a promising new therapeutic approach in CRC, which could ultimately lead to the better management of this deadly disease [30].

In the search for novel therapeutic agents that target the development and progression of cancer, natural products have become the focus of scientists because they have a multi-targeting potential and can overcome the disadvantages of monotherapy such as side effects [31] and drug resistance [32]. In fact, a number of plant-derived products have been identified that could interact, regulate, and thus target the TME [33]. The natural compound Calebin A is a non-curcuminoid [34] derived from the rhizome of medicinal turmeric (*Curcuma longa L. Zingiberaceae*), which is widely used in herbal medicinal applications [35,36,37]. Calebin A has been shown to exert anti-inflammatory and anti-tumor properties by the induction of apoptosis and modulating different signaling pathways [e.g., mitogen-activated protein kinase (MAPK), extracellular signal-regulated kinase (ERK), p38, Jun N-terminal kinase (JNK)] in gastric cancer and neurofibroma [38,39]. Several in vitro studies have revealed that Calebin A repressed the NF-κB signaling pathway in different cancer cell lines [37]. Recent studies have delineated that Calebin A possesses immense potential to suppress the tumor progression in CRC [25,40]. Calebin A suppressed not only pro-inflammatory cytokine TNF-β-induced NF-κB pathway activation, inhibiting proliferation, migration, and stimulated apoptosis in CRC cells [25,40], but also chemosensitized the CRC cells further towards 5-fluorouracil [40].

Since paracrine interaction in the TME plays a crucial role in stimulating the tumor progression process linked with apoptosis inhibition, in the present study we investigated the potential role of Calebin A in targeting the NF-κB signaling pathway to suppress the cross-talk in the TME and to stimulate apoptosis in CRC in a multicellular pro-inflammatory TME, in vitro.

## 2. Materials and Methods

### 2.1. Antibodies and Chemicals

Anti-phospho-p65-NF-κB, anti-p65-NF-κB, anti-matrix metalloproteinase 9 (MMP-9), anti-chemokine receptor type 4 (CXCR4), anti-cyclin D1, anti-survivin, anti-PARP, and anti-cleaved-Caspase-3 were purchased from R&D Systems (Heidelberg, Germany). Anti-Bcl-2 and anti-Bcl-xL were obtained from Santa Cruz Biotechnology (Santa Cruz, CA, USA). Rhodamine-coupled secondary antibodies for immunofluorescence were from Dianova (Hamburg, Germany). MTT reagent (3-(4,5-dimethylthiazol-2-yl)-2,5-diphenyltetrazolium bromide), 4′,6-diamidino-2-phenylindole (DAPI), BMS-345541, and anti-β-Actin were from Sigma-Aldrich (Taufkirchen, Germany). Calebin A was a kind gift from Sabinsa Corporation (East Windsor, NJ, USA) and was prepared as a 10.000 µM stock solution in DMSO. For the experiments, the Calebin A stock solution was further diluted in the cell culture medium and the final concentration of dimethyl sulfoxide (DMSO) did not exceed 0.1%.

### 2.2. Cells and Cell Culture Conditions

HCT116, a CRC cell line, and MRC-5, a normal human fibroblast cell line, were acquired from the European Collection of Cell Cultures (Salisbury, UK). CRC and MRC-5 cells were cultivated as monolayers under standard conditions (37 °C, 5% CO_2_) with whole-cell culture growth medium (10% fetal calf serum (FCS)) as described above [41] and passaged when cells reached 70–80% confluency. The human T-lymphocyte cell line (Jurkat cells) was obtained from the Leibniz Institute (DSMZ-German Collection of Microorganisms and Cell Cultures), and the cells were cultivated in suspension with a whole-cell culture growth medium [42].

### 2.3. Experimental Study Design

**Figure 1:**
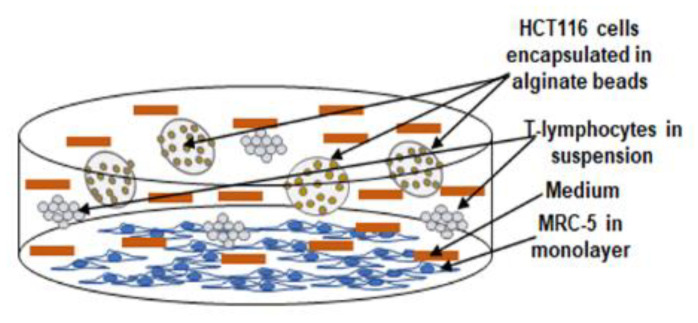
Schematic model showing the experimental setup of multicellular pro-inflammatory TME culture conditions.

In this study, we established a multicellular pro-inflammatory 3-dimensional (3D) TME similar to an in vivo TME to investigate the effect of Calebin A on the suppression of TME cross-talk (Figure 1).

For the "multicellular pro-inflammatory TME", MRC-5 cells (normal stromal fibroblasts) were seeded in a Petri dish (3000 cells/cm^2^) with a normal cell culture growth medium for 24 h to adhere to the bottom of the Petri dish. Next, 3D-alginate bead cultures of HCT116 CRC cells were established as described below. To create the "multicellular pro-inflammatory TME", HCT116 in 3D-alginate beads and T-lymphocytes (10.000/mL) was added to the Petri dishes containing the MRC-5 fibroblast cells and co-cultured in 3% FCS culture growth medium. For a "basal control" HCT116 CRC cells were cultivated in 3D-alginate beads alone. For the experiments, the multicellular pro-inflammatory TME cultures and HCT116 cultures of the basal control were cultivated either alone or in combination dose-dependently with Calebin A (1, 5 µM) or with BMS-345541 (5, 10 µM).

### 2.4. Alginate Culture

HCT116 CRC cells (1 × 10^6^/mL) were suspended in sterile alginate solution (2% in 0.15 M NaCl, stirred for 2 h at ambient temperature) and a 3D-alginate bead culture was prepared as described in detail [41,43]. Briefly, the HCT116 cell/alginate solution was added dropwise to a CaCl_2_ solution (100 mM). After a 10-minute polymerization of the drops to beads, the beads were washed 3 times with NaCl solution (0.15 M), once with a whole-cell culture medium (10% FCS) and before starting the experiments, the beads were incubated for 1 h with a serum-starved medium (3% FCS).

### 2.5. Vitality and Proliferation

The vitality and proliferation of HCT116 cells in alginate beads from TME cultures was investigated using the MTT test as described previously [27]. In short, HCT116 cells were retrieved from the alginate by dissolving for 15 min in sterile 55 mM sodium citrate solution. After continuous washing with Hank’s balanced salt solution, cells were suspended in modified MTT culture medium (without phenol red, without vitamin C, 3% FCS), 100 µL cell suspension/well distributed to a 96-well plate, and 10 μL MTT solution (5 mg/mL) added to each well. After 3 h incubation the reaction was blocked by adding 100 µL MTT-solubilization solution (10% Triton x-100/acidic isopropanol) to each well and samples incubated overnight at 37 °C. Finally, metabolically active cancer cells were determined by measuring the Optical Density at 550 nm (OD550) using a revelation 96-well multiscanner plate ELISA reader (Bio-Rad Laboratories Inc. Munich, Germany).

### 2.6. Colonosphere Formation and Invasion

The colonosphere formation and invasion capacity of HCT116 in 3D-alginate beads in TME culture were investigated as previously described [25]. For colonosphere formation, 25 microscopic fields were evaluated by light microscopy (Zeiss, Oberkochen Germany) and for colony formation, invaded and newly adhering colonies were stained with toluidine blue and quantified by counting all colonies under the light microscope (Zeiss, Oberkochen Germany) and the images were digitally stored.

### 2.7. Immunofluorescence

The “multicellular pro-inflammatory TME” described above was modified for immunofluorescence examination. HCT116 CRC cells were seeded in a monolayer on glass plates (5000/glass plate), MRC-5 fibroblasts were seeded separately (3000 cells/cm^2^) in Petri dishes, and cells were cultivated in a whole-cell culture growth medium (10% FCS) for 24 h to allow adherence. Multicellular pro-inflammatory TME cultures were established by placing HCT116-containing glass plates on a steel mesh bridge in the petri dishes containing the stroma fibroblasts and adding T-lymphocytes (20,000/mL) to the cultures. To allow the development of the TME, the cultures were cultured in a serum-starved medium for 24 h before starting the tests. The "basal control" held only HCT116 in the monolayer culture on glass plates (5000/glass plate). For the experiments, the multicellular pro-inflammatory TME cultures and basal control HCT116 cultures were either cultured alone or in combination dose-dependently with Calebin A (1, 5 µM) or with BMS-345541 (5, 10 µM). Immunofluorescence investigation of NF-κB expression in HCT116 CRC cells in TME cultures was performed as previously described [40]. In short, after 10 min methanol fixation, HCT116 were washed twice with Hank’s balanced saline solution and incubated overnight (4 °C) with primary antibodies (1:80) in a humid chamber. After additional washing with Hank’s balanced saline solution the samples were incubated for 2 h with rhodamine coupled secondary antibodies (1:100) and 15 min with DAPI to stain cell nuclei. Finally, the samples were embedded with Fluoromount (Sigma-Aldrich, Taufkirchen, Germany), the images were acquired with a Leica DM2000 microscope (Wetzlar, Germany) and stored digitally. Quantification of the expression of NF-κB and apoptotic nuclei were performed by counting 600–800 cells in 20 microscopic fields.

### 2.8. Western Blotting Investigations

Immunoblotting was performed on HCT116 beads from TME cultures as described in detail [41]. In short, HCT116 were retrieved from alginate beads by dissolving in 55 mM sodium citrate solution for 15 min and subsequent washing with Hank’s solution. Samples were lysed (50 mM Tris/HCl, pH 7.2/150 mM NaCl/(*v/v*) Triton X-100/1 mM sodium orthovanadate/50 mM sodium pyrophosphate/100 mM sodium fluoride/4 µg/mL pepstatin A/1 mM PMSF) for 30 min on ice to extract whole-cell proteins. Protein content was measured with the bicinchoninic acid system (Uptima, France) using bovine serum albumin (BSA) as standard, proteins reduced with 2-mercaptoethanol and total protein concentrations adjusted (500 ng per lane total protein). After separation of proteins with sodium dodecyl sulfate–polyacrylamide gel electrophoresis (SDS-PAGE), proteins were blotted onto a nitrocellulose membrane using a transblot apparatus (Bio-Rad, Munich). Membranes were incubated overnight (4 °C) with primary antibodies (1:10.000) and after subsequent washing, incubated for 2 h with alkaline-phosphatase coupled secondary antibodies (1:10.000). Finally, specific binding was detected using nitro blue tetrazolium and 5-bromo-4-chloro-3-indoyl-phosphate (VWR, Darmstadt, Germany) and bands quantified using the Quantity One program (Bio-Rad, Munich). β-Actin was used to normalize samples to control.

### 2.9. DNA-Binding Assay

Nuclear extracts of HCT116 CRC cells from multicellular pro-inflammatory TME cultures were prepared to further clarify the effect of Calebin A on p65-NF-κB binding to DNA. HCT116 cultured in multicellular pro-inflammatory TME or as a basal control were obtained from alginate, the cytoplasm was extracted with cytoplasm extraction buffer and the obtained nuclei were either left untreated and/or treated with Calebin A (1, 2, 5, 10 µM) for 30 min in a dose-dependent manner. In an additional approach, HCT116 cells were cultured in multicellular pro-inflammatory TME cultures or as basal controls, and the extracted nuclei were either left untreated and/or treated with Calebin A (5 µM), DTT (5 mM) alone or in combination for 1 h. Finally, nuclear extracts were prepared, proteins separated by SDS-PAGE and blotted on nitrocellulose membranes as described above.

### 2.10. Statistical Evaluation

A Wilcoxon–Mann–Whitney test was used for statistical evaluation. The samples were presented as mean ± SD or SEM and compared by one-, two- or three-way ANOVA using SPSS Statistics if the normality test was passed (Kolmogorov–Smirnov test). A significant difference was considered with a *p*-value of < 0.05.

## 3. Results

In the current study, we investigated the capacity of Calebin A in targeting the NF-κB signaling pathway and the NF-κB-regulated biomarker gene expression promoted by multicellular pro-inflammatory TME (Figure 1).

### 3.1. Calebin A Suppresses Proliferation Promoted by TME Cultures in 3D-Alginate CRC Cells

**Figure 2:**
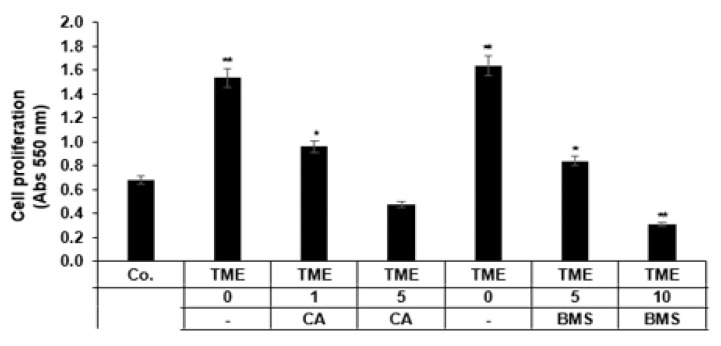
Effect of Calebin A (CA) or specific IKK inhibitor BMS-345541 on the proliferation of CRC cells: Serum starved HCT116 cells in alginate beads from the basal control and multicellular TME cultures were treated as described in Materials and Methods. Cell proliferation was analyzed with the MTT method. All experiments were performed at least three times. *p* < 0.05 (*) and *p* < 0.01 (**) indicate a significant difference compared to the control group.

Since the pro-inflammatory transcription factor NF-κB is involved in cell survival and cell proliferation [44,45], we investigated the effect of Calebin A or BMS-345541 on the proliferation of HCT116 CRC cell lines using the MTT method. We used BMS-345541, a specific inhibitor of the IKK complex, which can specifically suppress NF-κB activation promoted by various stimuli [46]. HCT116 cells were left untreated or treated with Calebin A, or BMS-345541 and cell proliferation was evaluated by MTT assay as described in Materials and Methods. Multicellular pro-inflammatory TME enhanced the proliferation of HCT116 cells significantly compared to the control (Figure 2). In contrast, Calebin A, similar to BMS-345541, suppressed TME-enhanced proliferation of CRC cells in 3D alginate tumor cultures in a dose-dependent fashion. TME by itself increased the cell proliferation by more than double compared to the basal control, enhancing it by 130%. In contrast, the treatment of TME cultures with Calebin A or BMS-345541 significantly reduced the proliferation of CRC cells in a dose-dependent manner, compared to the TME control cultures, by around 41% and 59% in Calebin A (1, 5 µM) and by 49% and 81% in BMS-345541 (5, 10 µM), respectively (*p* < 0.05) (Figure 2). Altogether, these results suggest that the multicellular pro-inflammatory TME, which significantly favored the proliferation of HCT116 CRC, and the IKK inhibitor (BMS-345541), similar to Calebin A, blocked the stimulation effects of the TME.

### 3.2. Calebin A Suppresses Invasion and Colony Formation Ability, Promoted by TME in 3D-Alginate CRC Cells

**Figure 3:**
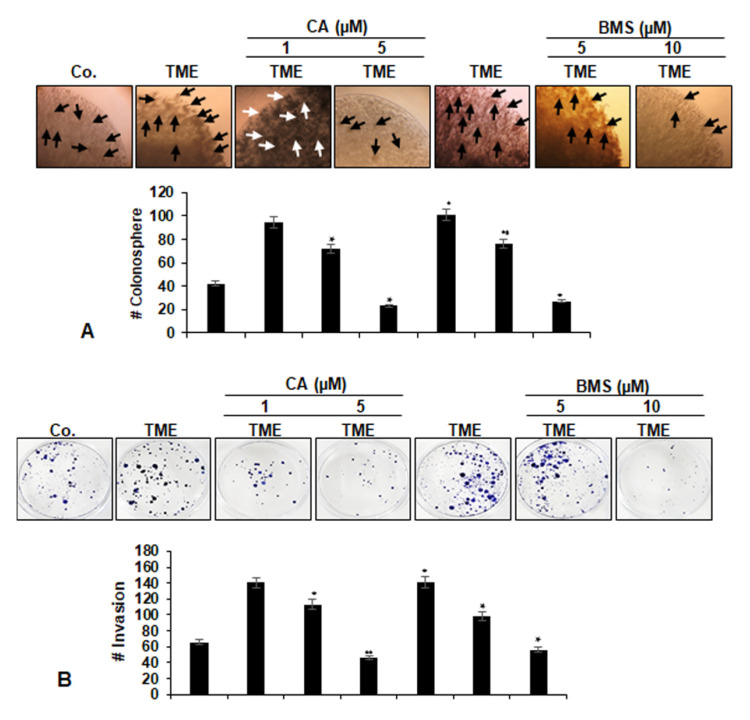
Effects of Calebin A (CA) or specific IKK inhibitor BMS-345541 on colonosphere formation and invasion in of CRC cells: Serum starved HCT116 cells in alginate (stars) were treated as described in Materials and Methods. Colonosphere formation (**A**) and invasion (**B**) were analyzed by light microscopy after 10 days. All experiments were performed at least three times. The number of colonospheres (arrows) was quantified by counting 25 different microscopic fields, and the number of attached colonies was quantified in each well. *p* < 0.05 (*) and *p* < 0.01 (**) indicate a significant difference compared to the control group. Magnification A: × 24, bar = 0.2 mm.

Colony formation and invasion studies, which more accurately reflect the situation in vivo, were carried out as described in Materials and Methods. As demonstrated in Figure 3A,B, multicellular pro-inflammatory TME markedly promoted tumor cell migration and colony formation in CRC alginate cultures compared to the basal control. In contrast, Calebin A, similar to BMS-345541 dose-dependently blocked colony formation and invasion of HCT116 cells in alginate TME cultures. At the dose 5 µM Calebin A and 10 µM, BMS-345541 colony formation was decreased by 72% and by 57%, respectively, compared to the untreated TME control (Figure 3A,B).

### 3.3. Calebin A Decreases TME-Induced Activation and Nuclear Translocation of p65-NF-κB in CRC Cells 

**Figure 4:**
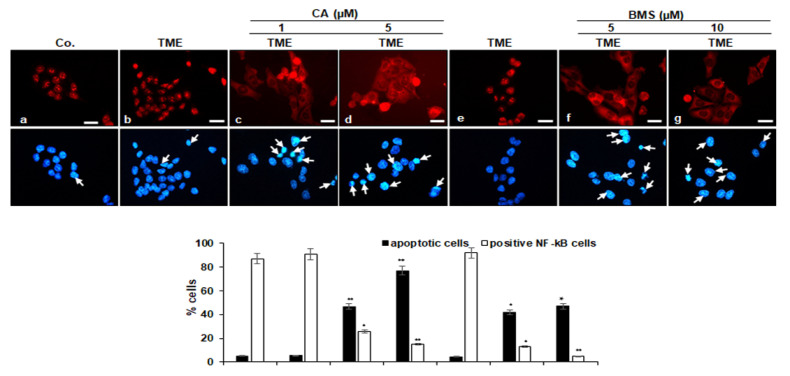
Effect of Calebin A (CA) or specific IKK inhibitor BMS-345541 on activation and nuclear translocation of p65-NF-κB in CRC cells: Serum-starved HCT116 cells in a monolayer culture from multicellular pro-inflammatory TME cultures were treated as described in Materials and Methods, stained for p65 subunit of NF-κB by immunofluorescence and counterstained with DAPI. Magnification 600×; scale bar = 30 mm. All experiments were performed at least in triplicate and quantification of positively labelled p65-NF-κB-nuclei and apoptotic nuclei (white arrows) were performed by counting 600–800 cells from 20 different microscopic fields. Values were compared with the control and *p* < 0.05 (*), *p* < 0.01 (**) were considered statistically significant.

We further investigated whether Calebin A modulates the TME-promoted nuclear translocation of p65-NF-κB. It is known that cytokines in the pro-inflammatory TME induce the activation and phosphorylation of p65-NF-κB, which is necessary for its transcriptional activity so that after phosphorylation the p65 subunit is translocated into the nucleus of cells [47]. In the untreated multicellular pro-inflammatory TME cultures, HCT116 cells showed strong nuclear labeling for p65-NF-κB around 91% and slight cytoplasmic labeling (Figure 4), compared to HCT116 cells in the basal control (87%) with a slightly lower expression of p65-NF-κB. In contrast, Calebin A in the multicellular pro-inflammatory TME cultures inhibited distinctly nuclear staining and nuclear translocation of p65-NF-κB in CRC cells in a dose-dependent fashion by around 71% and 84% (Calebin A 1, 5 µM) compared to the control TME cultures (Figure 4).

Since phosphorylation and degradation of IκBα require activation of IκB kinase (IKK), we investigated the capacity of BMS-345541, a specific IKK blocker, on TME-promoted IKK activity. Interestingly, treatment of HCT116 cells in multicellular TME with BMS-345541 significantly inhibited p65-NF-κB nuclear translocation, depending on the dose, by around 85% and 95% (5, 10 µM) compared to the control TME cultures (Figure 4). The additional investigation of apoptosis stimulation with DAPI staining revealed minimal apoptotic morphological changes in untreated cells in multicellular TME (5%). In accordance with the phospho-p65 translocation data, treatment with Calebin A or BMS-345541 alone significantly increased apoptotic morphological changes in alginate HCT116 cells in multicellular pro-inflammatory TME with 50% to 70% apoptotic cells in HCT116 treated with Calebin A (1, 5 µM) and with around 50% apoptotic cells in HCT116 treated with BMS-345541 (5, 10 µM) (Figure 4). These findings show that the IKK is an important regulating upstream kinase and is essential for the cytokine-NF-κB-mediated axis signaling pathway.

### 3.4. Calebin A Represses TME-Induced NF-κB Activation and NF-κB-Dependent Gene End-Products Involved in Cell Proliferation, Invasion, and Apoptosis in CRC Cells

**Figure 5:**
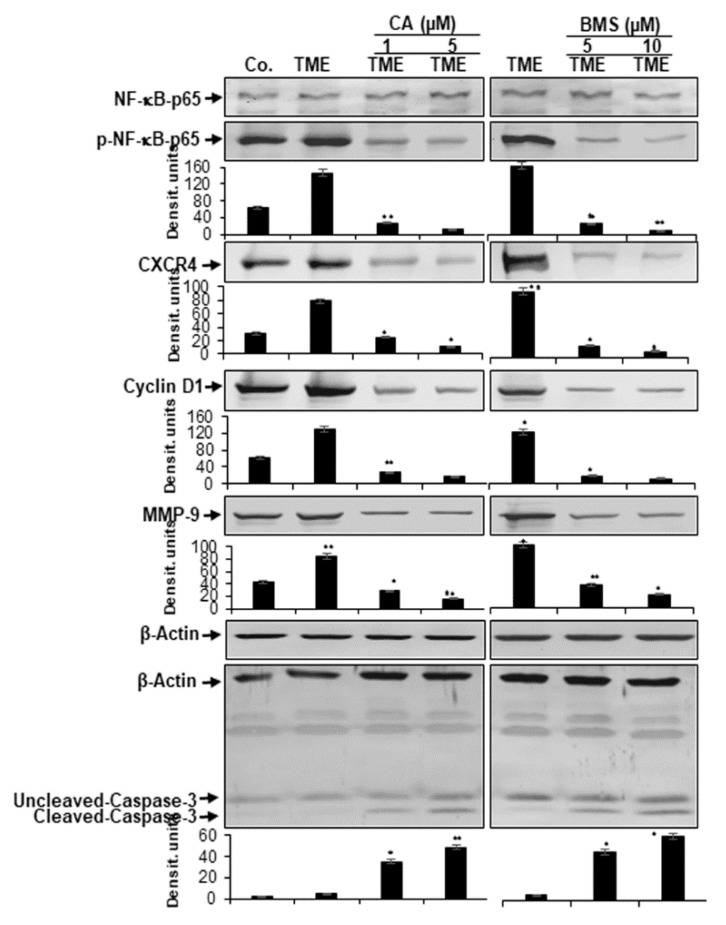
Effect of Calebin A (CA) or specific IKK inhibitor BMS-345541 on activation of NF-κB and NF-κB-regulated gene end-products in CRC cells: Serum-starved HCT116 cells in alginate cultures from multicellular pro-inflammatory TME cultures were treated as described in Materials and Methods. Immunoblotting of whole-cell lysates from HCT116 was performed for anti-p65-NF-κB, anti-phospho-p65-NF-κB, anti-MMP-9, anti-CXCR4, anti-Cyclin D1, and anti-cleaved-caspase-3. β-actin served as an internal loading control in all experiments. For densitometric evaluation, results are compared to control and *p* < 0.05 (*), *p* < 0.01 (**) were considered statistically significant.

It has been reported that cytokines and pro-inflammatory TME promote the phosphorylation of NF-κB and the expression of proliferative (cyclin D1), metastatic (MMP-9, CXCR4) tumor-promoting proteins and inhibit apoptosis (caspase-3), which have a p65-NF-κB binding site in their promoters [48,49,50,51,52,53]. The 3D-alginate cultures of HCT116 cells in multicellular TME were left untreated, or treated with Calebin A or BMS-34554, as described in the Materials and Method section. It was investigated whether Calebin A blocks the pro-inflammatory multicellular TME-induced activation of p65-NF-κB and the expression of the above-mentioned p65-NF-κB-dependent proteins. The results of the Western blot analysis showed that pro-inflammatory multicellular TME up-regulated the expression of all these proteins (p65-NF-κB, cyclin D1, MMP-9 and CXCR4), and down-regulated activated caspase-3. However, Calebin A, similar to IKK inhibitor (BMS-345541), suppressed them (p65-NF-κB, cyclin D1, MMP-9 and CXCR4) and up-regulated activated caspase-3 in a dose-dependent fashion, compared with those from the TME control group (Figure 5). These findings provide evidence for the important capacity of Calebin A in suppressing the activation of TME-promoted NF-κB and the expression of NF-κB-dependent gene products. In summary, these results suggest that one main and important TME-pathway promotes tumorigenesis in CRC cells by inducing the NF-κB signaling pathway. In addition, the anti-tumorigenic impact of Calebin A is, at least in part, mediated by the up-stream suppression of this pathway, similar to the inhibition of the NF-κB signaling pathway by BMS-345541.

### 3.5. Calebin A Reduces TME-Induced NF-κB-Dependent Anti-Apoptotic Gene Biomarkers in CRC Cells

**Figure 6:**
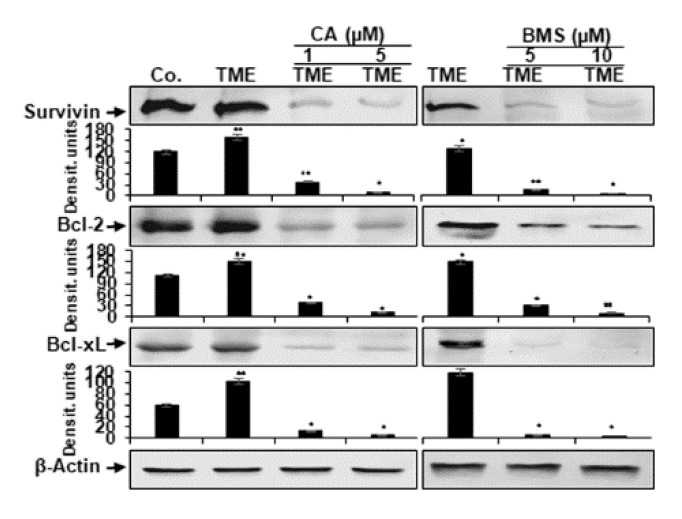
Effect of Calebin A (CA) or specific IKK inhibitor BMS-345541 on the expression of anti-apoptotic gene products in CRC cells: Serum-starved HCT116 cells in alginate cultures from multicellular pro-inflammatory TME cultures were treated as described in Materials and Methods. Immunoblotting of whole-cell lysates from HCT116 was performed for anti-survivin, anti-Bcl-2, and anti-Bcl-xL in HCT116 cells. Densitometric values were compared with the control and *p* < 0.05 (*), *p* < 0.01 (**) were considered statistically significant. β-actin was used as an internal loading control.

It is known that the pro-inflammatory transcription factor NF-κB promotes and stimulates the expression of anti-apoptotic biomarkers (tumor-promoting proteins) such as survivin, Bcl-2, and Bcl-xL [54,55,56,57,58,59,60,61]. Therefore, we investigated whether Calebin A can modulate the pro-inflammatory, multicellular TME-promoted expression of the aforementioned NF-κB-regulated anti-apoptotic gene biomarkers. We found that pro-inflammatory multicellular TME induces the expression of all the above-mentioned anti-apoptotic biomarkers. Conversely, Calebin A, similar to the IKK inhibitor (BMS-345541), represses pro-inflammatory multicellular TME-induced expression of these cancer-promoting proteins in a dose-dependent fashion (Figure 6).

### 3.6. Calebin A Disturbs the Direct Interaction of NF-κB with DNA in CRC Cells

**Figure 7:**
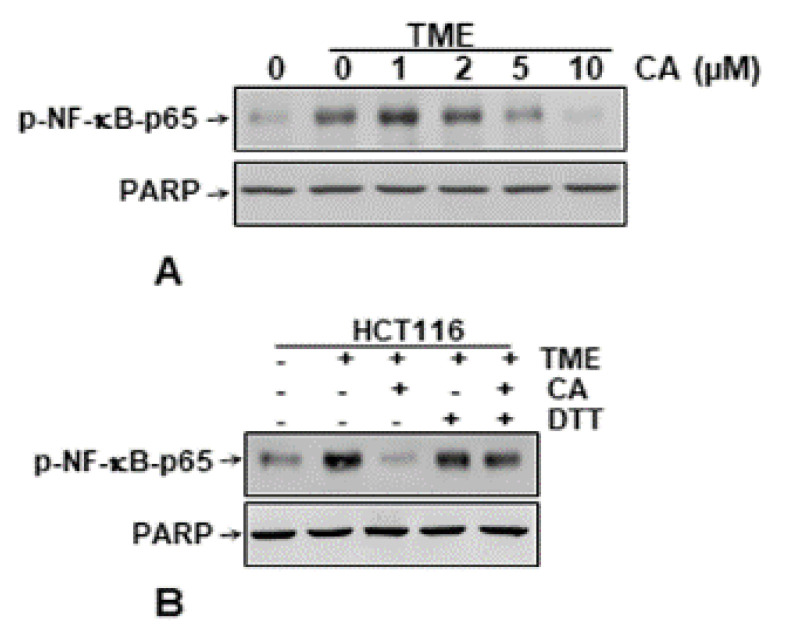
Direct effect of Calebin A (CA) on NF-κB binding to DNA in CRC cells: Serum-starved HCT116 cells in alginate cultures from multicellular pro-inflammatory TME cultures were treated as described in Materials and Methods. **A**: Isolated nuclei from basal control HCT116 cells or HCT116 cells from multicellular TME, incubated with the indicated concentrations of Calebin A for 30 min, nuclear extracts were prepared and then immunoblotted for p65-NF-κB activation by Western blotting. PARP served as an internal loading control in all experiments. **B**: Isolated nuclei from HCT116 basal control or multicellular TME, incubated for 30 min with 5 µM Calebin A in the presence or absence of 5 mM DTT, nuclear extracts were prepared and then probed for p65-NF-κB binding to DNA by Western blotting. PARP served as an internal loading control in all experiments.

It has been previously reported that some specific NF-κB blockers suppress the activation of the NF-κB pathway by interaction and modulation of NF-κB-subunits proteins, thereby inhibiting its interaction with DNA [40,62,63,64]. We analyzed whether Calebin A is able to trigger the inhibition of NF-κB activation in CRC cells in multicellular pro-inflammatory TME by a related pathway. To determine this, we incubated extracted nuclei from multicellular pro-inflammatory TME HCT116 cells with or without Calebin A as described in Materials and Methods. As shown in Figure 7**A**, Calebin A significantly blocked NF-κB interaction with DNA in a dose-dependent fashion. Since the cysteine groups at the 38 position in the p65 subunit of NF-κB are mainly associated with their attraction to DNA, we performed the test for the reduction in cysteine groups by DTT in the presence or absence of Calebin A, as described in the section Materials and Methods. We found that the inhibition capacity of Calebin A on the p65-NF-κB binding to the DNA was reversed by DTT in HCT116 nuclei, underlining that Calebin A specifically modulates the binding of p65-NF-κB to DNA (Figure 7**B**). Collectively, these findings suggest that the modulation of this essential binding may be one of the most critical molecular signaling steps of multi-targeted Calebin A, as it inhibits p65-NF-κB stimulation.

## 4. Discussion

**Figure 8:**
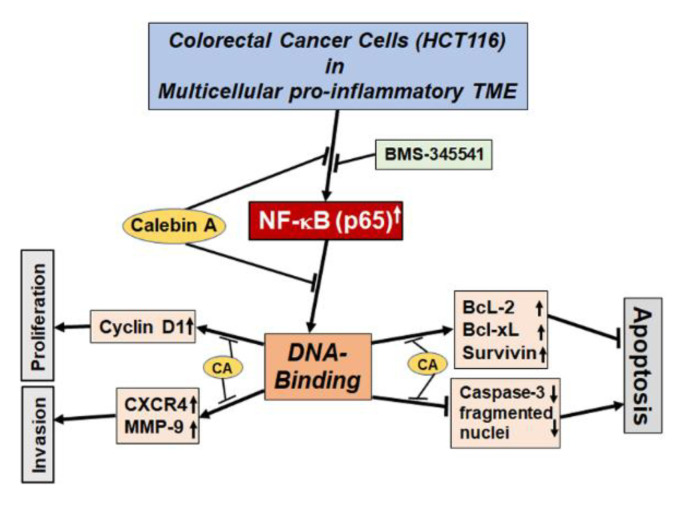
Modulatory impacts of Calebin A on the NF-κB signaling pathway in the multicellular TME.

In this study, we evaluated the molecular pathway by which Calebin A exerts its anti-inflammatory, anti-proliferative, and anti-tumor activity in a multicellular pro-inflammatory TME. We could show that Calebin A suppresses the master pro-inflammatory transcription factor NF-κB by inhibiting phosphorylation, and its translocation into the nucleus, which is promoted by the TME. Furthermore, Calebin A blocked the interaction of p65-NF-κB with DNA. In this way, Calebin A down-modulated NF-κB-promoting gene end-products involved in invasion, proliferation, and anti-apoptosis, ultimately triggering the induction of apoptosis, which was down-regulated by the TME (Figure 8).

In the present study, we have developed a novel 3D TME culture-system consisting of tumor cells (3D-alginate), T-lymphocytes, and fibroblasts to better understand and mimic the in vivo heterogeneous pro-inflammatory TME (Figure 1). In fact, it has been reported that fibroblasts are the main active cells of the tumor stroma in TME and are involved in promoting tumor progression by secreting tumor stimulating proteins, extracellular matrix, and enzymes [65,66]. Here, we evaluated, for the first time, the suppression effect of Calebin A on the NF-κB signaling and NF-κB-regulated cellular responses in detail on HCT116 CRC cells in alginate cultures in this TME. It is well established that NF-κB acts as a master regulator of inflammation and is closely associated with inflammatory reactions and chronic diseases, including cancer and TME [15,20,67,68]. Moreover, it has been shown that the various inflammatory responses stimulate the interaction of tumor cells and the stroma in the TME [11,24], however, the cell-specific and inflammation-induced molecular pathways that allow cancer cells to proliferate and metastasize in the TME are not fully described. In the TME, the constitutive activation of NF-κB promotes the survival and proliferation of tumor cells and stimulates the inappropriate activation of immune cells, which leads to the escape of tumor cells from apoptotic mechanisms [11]. It is well documented that the TME is infiltrated by a wide range of immune cell populations [69], and recent data suggest that immune cell infiltrates may also affect prognosis in CRC [70]. However, whether immune cell infiltrates in the TME could have a poor or improved prognosis remains controversial [11]. Interestingly, it has been suggested that the activation of the NF-κB signaling pathway plays a key role not only in the activation of signal transduction in cancer cells, but also in the recruitment of infiltrating leukocytes in the TME [71]. In fact, NF-κB enhances cross-talk with the TME containing cells and the stromal and inflammatory cells, through the up-regulation of pro-inflammatory cytokines, such as TNF-α [72]. In acute lymphoblastic leukemia (T-ALL) of T cells, the inhibition of the NF-κB signaling pathway could suppress tumor growth both in the in vitro and the in vivo settings [73]. Interestingly, in a mouse colitis model it was shown that the deletion of IKK in intestinal epithelial cells down-regulated expression of pro-inflammatory cytokines, highlighting the crucial role of inflammation in supporting cancer and indicating that specific blocking of the pro-inflammatory IKK/NF-κB pathway may be an attractive and promising therapeutic target [74]. On the basis of our findings, we suggest that this 3D-multicellular pro-inflammatory TME model could also be useful for the testing and screening of various types of tumor, therapeutic agents/drugs, and for the investigation of different therapeutic targets. 

In a similar way to BMS-345541 (a specific pharmacological highly selective inhibitor of IKK) [46] Calebin A displayed its cytotoxic and anti-proliferative activities in the multicellular pro-inflammatory TME. It was evidenced through MTT assay and confirmed by colonosphere and invasion assays. In addition, these specific anti-tumor actions of Calebin A further correlated with inhibition of cell metastatic (CXCR4, MMP-9) and proliferative (cyclin D1) biomarkers, all of which are known to have a NF-κB binding site in their promoters, thus promoting their transcription. These results are consistent with our previous reports with various other natural products such as turmeric [25,40,75] and resveratrol [26,27,76,77]. Moreover, Calebin A, similar to BMS-345541, reduced the multicellular pro-inflammatory TME-stimulated phosphorylation and translocation of the p65 subunit of NF-κB from the cytoplasm to the nucleus, suggesting that Calebin A, which possesses pro-apoptotic properties, has the potential to prevent tumor invasion and proliferation, at least in part, by up-stream targeting the IKK-NF-κB signaling pathway. In fact, it has been reported that the down-modulation of p65-NF-κB in cancer cells at different levels of the signaling pathway increases the benefits for therapeutic approaches [27,78]. To the best of our knowledge, this is the first study to date to show the anti-inflammatory and anti-tumor impacts of Calebin A on multicellular pro-inflammatory TME, at least in part through targeting the suppression of the NF-κB signaling pathway. 

We also found that Calebin A blocked the direct interaction between p65-NF-κB and DNA, indicating its specific targeting and modulation capacity of the p65-NF-κB protein. Previous studies have demonstrated that p65-NF-κB subunits with its cysteine^38^ residue are responsible for the interaction between NF-κB and DNA [79]. Blocking this specific interaction by Calebin A could be inhibited by a reducing agent, such as DTT, suggesting that Calebin A may modulate cysteine residues in the p65-NF-κB subunit. In fact, this finding is consistent with a large body of evidence that has previously shown that numerous active ingredients such as caffeic acid, phenethyl ester [63], plumbagin [64], thymoquinone [80], sesquiterpene lactone parthenolide [79], and Calebin A have a similar action [37,40]. However, this result shows for the first time that Calebin A inhibits multicellular pro-inflammatory TME-promoted NF-κB stimulation by direct blocking between p65-NF-κB and DNA, and this may be one of the most essential molecular mechanisms of Calebin A, as it blocks p65-NF-κB stimulation. Based on our results, we assume that specific NF-κB activation in tumor cells was precisely associated with the development of this multicellular pro-inflammatory TME by promoting proliferation, colonosphere formation, and invasion ability. Furthermore, these results clearly indicate a functional relationship between the NF-κB signaling pathway and the inflammation-induced aggressiveness of CRC.

We found that multicellular pro-inflammatory TME promoted NF-κB activation, which in turn mediated the inhibition of apoptosis by the expression of various anti-apoptotic gene biomarkers, which indicate a driving function of NF-κB in the TME-stimulated augmentation of CRC cell malignancy. In addition, we have shown that Calebin A, similar to BMS-345541, decreased the expression of survivin, Bcl-2, and Bcl-xL proteins. As a matter of fact, it has been previously reported that other natural compounds suppress the anti-apoptotic proteins in human CRC cells [81,82]. Furthermore, we have shown that Calebin A, similar to BMS-345541, induced apoptosis through the activation of the apoptotic NF-κB-regulated gene biomarker, caspase-3 and the increasing of pyknosis, chromatin condensation, and apoptotic body formation. Based on these data, we could clearly demonstrate that this mixed heterogeneous pro-inflammatory TME with NF-κB up-regulation is a suitable functional paracrine transmitter of CRC tumorigenesis in vitro.

Overall, our findings illustrated that Calebin A significantly suppressed NF-κB activation and NF-κB-promoted anti-apoptotic biomarkers, making it a potentially effective inhibitor of inflammation, proliferation, invasion, and survival of cancer cells.

## 5. Conclusions

To conclude, our results indicate a crucial positive interaction between stroma cells and CRC cells in this multicellular pro-inflammatory TME, which is essential for the induction of CRC cells tumor progression, proliferation, and invasion. Furthermore, Calebin A has a significant anti-inflammatory potential and it down-regulates multicellular TME-induced tumor malignancy by specific targeting the p65-NF-κB signaling pathway, leading to suppressed proliferation and induction of apoptosis. Our results underline further that the NF-κB signaling pathway may be a potential therapeutic target for CRC treatment. Further studies on Calebin A could provide important information for potential applications within combination therapies in the management of cancer and other chronic diseases.
